# 5/6 nephrectomy induces different renal, cardiac and vascular consequences in 129/Sv and C57BL/6JRj mice

**DOI:** 10.1038/s41598-020-58393-w

**Published:** 2020-01-30

**Authors:** Mouad Hamzaoui, Zoubir Djerada, Valery Brunel, Paul Mulder, Vincent Richard, Jérémy Bellien, Dominique Guerrot

**Affiliations:** 1 Normandie Univ, UNIROUEN, INSERM U1096, FHU REMOD-VHF, 76000 Rouen, France; 2 Nephrology Department, Rouen University Hospital, Rouen, France; 3 Pharmacology Department, Reims University Hospital, Reims, France; 4 Biochemistry Department, Rouen University Hospital, Rouen, France; 5 Pharmacology Department, Rouen University Hospital, Rouen, France

**Keywords:** Renovascular hypertension, End-stage renal disease

## Abstract

Experimental models of cardiovascular diseases largely depend on the genetic background. Subtotal 5/6 nephrectomy (5/6 Nx) is the most frequently used model of chronic kidney disease (CKD) in rodents. However, in mice, cardiovascular consequences of 5/6 Nx are rarely reported in details and comparative results between strains are scarce. The present study detailed and compared the outcomes of 5/6 Nx in the 2 main strains of mice used in cardiovascular and kidney research, 129/Sv and C57BL/6JRj. Twelve weeks after 5/6 Nx, CKD was demonstrated by a significant increase in plasma creatinine in both 129/Sv and C57BL/6JRj male mice. Polyuria and kidney histological lesions were more pronounced in 129/Sv than in C57BL/6JRj mice. Increase in albuminuria was significant in 129/Sv but not in C57BL/6JRj mice. Both strains exhibited an increase in systolic blood pressure after 8 weeks associated with decreases in cardiac systolic and diastolic function. Heart weight increased significantly only in 129/Sv mice. Endothelium-dependent mesenteric artery relaxation to acetylcholine was altered after 5/6 Nx in C57BL/6JRj mice. Marked reduction of endothelium-dependent vasodilation to increased intraluminal flow was demonstrated in both strains after 5/6 Nx. Cardiovascular and kidney consequences of 5/6 Nx were more pronounced in 129/Sv than in C57BL/6JRj mice.

## Introduction

Chronic kidney disease (CKD) is a major health problem, with a worldwide prevalence of 13%^1^. A strong association has been demonstrated between the decrease in glomerular filtration rate (GFR), the risk of developing functional and structural disorders of the heart and cardiovascular (CV) events^2–4^. Accordingly, CV disease represents the leading cause of mortality in this population. Multiple mechanisms lead to the CV consequences of CKD, and endothelial dysfunction is considered as a cornerstone in this setting^5,6^. Due to limited pathophysiological understanding, a large amount of research is currently dedicated to the deleterious reciprocal interactions of heart and kidney diseases, within the 5 types of cardiorenal syndrome^7^. Different models of CKD have been developed in rodents, including kidney mass reduction by 5/6 nephrectomy (5/6 Nx), DOCA salt nephropathy, unilateral ureteral obstruction, oxalate-induced CKD, adenine-induced CKD and genetic mutation of *Col4A3*^8–12^, but these models do not fully reproduce the CV consequences of CKD observed in humans, including coronary heart disease. 5/6 Nx has been widely used in rats^13,14^ because of the persistent decrease in GFR, proteinuria, glomerular sclerosis and hypertension induced, contributing to the major interest of this model^15^. To date, several factors, including surgical difficulties, have limited the use of 5/6 Nx in mice^16^. This is of critical importance in particular because transgenic mice, most commonly developed on a C57BL/6JRj background, currently represent a key experimental tool. In the literature reporting results of 5/6 Nx in mice, strain-dependent differences appear regarding hypertension, albuminuria, kidney function, and kidney histological lesions^17^. Irrespective of the genetic background, renal, cardiac and vascular consequences of 5/6 Nx in mice are generally the subject of focused studies, and their occurrence and severity are therefore poorly characterized together.

In this context, the aim of this study is to comparatively describe the consequences of 5/6 Nx in the two commonly used mouse strains C57BL/6JRj and 129/Sv, with a specific focus on structural and functional disorders of the cardiovascular system.

## Methods

### Animal models

All experiments were carried out in C57BL/6JRj and 129/Sv mice (n = 20 per strain), aged 8 weeks and weighing between 20 and 26 g (directly bought from Janvier laboratory, Le Genest Ste Isle, France without breeding within our facility), in accordance with the standards and ethical rules, and with approval by the national animal ethics committee (CENOMEXA C2EA-54). To limit the number of animals used, and variability related to hormonal issues which play an important role in CV disorders beyond the scope of strain-dependent differences, only male mice were studied. The surgical procedures were performed by a single experienced operator in order to ensure reproducibility. Briefly, mice were anaesthetized by intraperitoneal injection of ketamine (100 mg/kg) (Ketamine® 1000, Virbac France) + Xylazine (10 mg/kg) (Rompun® 2%, Bayer France). After shaving and disinfection with povidone (10% Betadine® dermal, Meda), a left laparotomy was performed to expose the left kidney. First, in order to preserve the adrenal gland, it was separated from the left kidney without harming its vascularization, followed by dissection of the perihilar adipose tissue. After detecting the bifurcation of the kidney artery at the hilum, using a non-absorbable polypropylene 8-0 thread, 6.5-mm needle (Prolene® 8-0, Ethicon), the upper limb of the kidney artery was ligated and verified by the subsequent homogeneous discoloration of the upper half of the left kidney. The identification and ligation of the upper branch of the kidney artery was easier in C56BL/6JRj than in 129/Sv mice, since the division of the artery was more proximal in C56BL/6JRj as shown in Suppl Fig. 1.

**Figure 1:**
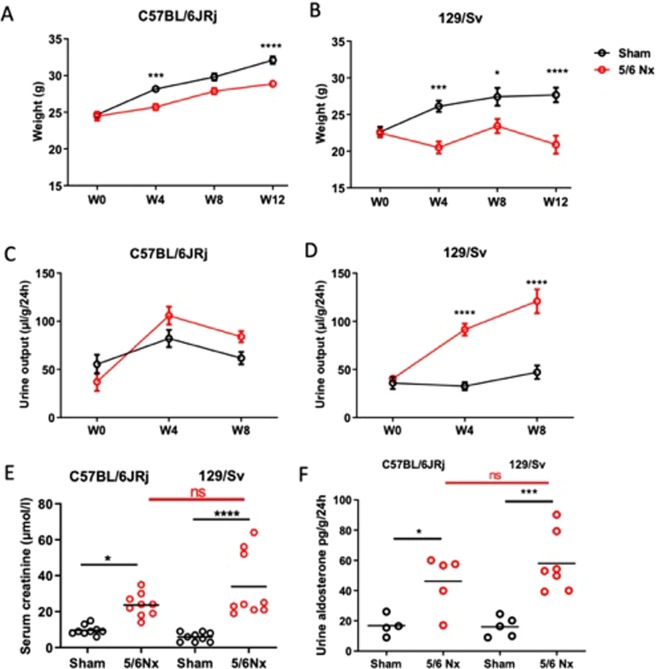
Evolution of body weight (**A**,**B**) and urine output (**C**,**D**) from week 0 (W0; 5/6 Nx) to W12 (sacrifice) in sham-operated and 5/6 Nx C57BL/6JRj (**A**,**C**) and 129/Sv (**B**,**D**) mice (n = 10 per group). *p < 0.05: sham *vs*. 5/6 Nx, **p < 0.01: sham *vs*. 5/6 Nx, ***p < 0.001: *vs*. 5/6 Nx. Plasma creatinine at week 12 (**E**) and aldosteronuria (**F**) in sham-operated and 5/6 Nx C57BL/6JRj and 129/SV mice (n = 8–9 per group). *p < 0.05: sham *vs* 5/6 Nx, ***p < 0.001: sham *vs* 5/6 Nx.

Then, using a cauterizer (Thermal Cautery Unit®, Geiger), the lower kidney pole was cauterized with an estimated depth of 1 mm, avoiding the hilum (Fig. 1), and then sutured using a non-absorbable 4-0 polyamide thread (Ethilon® 4-0, Ethicon). The entire surgical procedure was performed under a binocular magnifying glass (magnification x2). One week after the left partial nephrectomy, a total right nephrectomy was performed following the same steps described above, excepted that the hilum which was fully ligated and the right kidney removed. Control groups were subjected to a sham operation, undergoing a simple laparotomy. The animals received post-operative analgesia consisting in a subcutaneous injection of buprenorphine (0.05 mg/kg) (Buprecare®, Axience). Mice were sacrificed 12 weeks (W12) after total right nephrectomy.

### Blood pressure

Non-invasive measurements of systolic blood pressure (SBP) were performed by tail cuff plethysmography (CODA, Kent Scientific Corporation), before (W0), 4 (W4) and 8 (W0) weeks after surgery. These measurements were performed in conscious and trained mice, and consisted in 2 series of 10 cycles of measurements.

### Urine parameters

Urine was collected over 24 hours in metabolic cages at W0, W4 and W8 for evaluation of urine output. In addition, urine albumin was determined at W8 using an ELISA kit (Albuwell M, Exocell). Furthermore, urine aldosterone concentration was evaluated at W8 by ELISA (Aldosterone ELISA Kit, Enzo) and expressed as pg/g body weight/24 h.

### Plasma creatinine

At W12, mice were anesthetized (Xylazine + Ketamine as described previously), blood samples were collected and centrifuged. Plasma creatinine concentration was monitored by enzymatic method (CREP2, Roche Diagnostics).

### Cardiac function and dimensions

Ten weeks after the right nephrectomy mice were anesthetized (isoflurane 1 to 2%), LV size and function were assessed with a Vivid 7 ultrasound device (GE medical)^18^. The heart was imaged in 2-D mode (parasternal short-axis view). Ejection fraction (EF) was calculated as EF (%) = ((LVDA – LVSA)/LVDA) × 100, where LVDA and LVSA represent LV diastolic and systolic areas, estimated from end-diastolic and end-systolic diameter (EDD and ESD). Doppler measurements were also performed at the tip of mitral leaflets to determine diastolic filling profiles (apical 4-chamber view), to determine peak early (E) and late (A) mitral inflow velocities, and calculate E/A ratio, as an index of LV diastolic function.

### Vascular function

Endothelial function was first assessed on small vessel myographs and arteriographs as described previously^19,20^. After blood sampling, the mesentery was first removed and then placed in oxygenated Krebs buffer at 4 °C. A 1.5–2.0 mm segment of first order of mesenteric resistance artery segment was mounted on a myograph (DMT, Aarhus, Denmark). After normalization, endothelium-dependent relaxation to acetylcholine (10^−9^ to 3.10^−5^ mol/L) and endothelium-independent relaxation to sodium nitroprusside (SNP) (10^−9^ to 3.10^−5^ mol/L) were performed in segments pre-contracted with Phenylephrine (Phe: 10^−5^ mol/L). A 2-3 mm segment of third mesenteric resistance artery segment was mounted on an arteriograph (DMT, Denmark). The dilatory response to stepwise increases in intraluminal flow (from 3 to 100 μL/min) was assessed in vessels pre-constricted with Phe (10^−5^ mol/L). Only vessels which increased their constriction by more than 40% with Phe were included in the experiments.

### Kidney and cardiac histology

In 5/6 Nx mice, the remnant kidney was removed at sacrifice. Kidneys were harvested and decapsulated. Kidney histological lesions were analyzed after Masson’s staining. Briefly, the kidneys were fixed for 24 hours in 4% formalin and embedded in paraffin after conventional processing. Three-μm slices were thereafter stained with Masson’s trichrome solution. The slides were independently examined on a blinded basis, using a 0 to 4 injury scale for the level of interstitial inflammation, interstitial fibrosis and glomerulosclerosis at magnification x20 (0: no damage; 1: < 25% of kidney damaged; 2: 25–50% of kidney damaged; 3: 50–75% of kidney damaged; 4: 75–100% of kidney damaged). Tubular lesions were analyzed at magnification x10. Vascular thickening and vascular fibrosis were analyzed at magnification x40. The upper half of the kidney was not analyzed since it was ischemic in 5/6 Nx mice. The heart was harvested and weighed. A standardized section of the left ventricle (LV) was frozen for determination of LV fibrosis, using 8-µm thick histological slices, previously stained with Sirius Red. For each heart, 12–15 consecutive images were taken at x10 magnification. Fibrosis area was determined by blinded automated morphometric analysis and expressed as a mean percentage of the total area analyzed (Image J software).

### Statistical analyses

Statistical analysis was performed with Prism software (GraphPad Prism 5.00.288). Data were expressed as mean values ± SEM. The normality of the data and homogeneity of variances were respectively assessed by Shapiro-Wilk and Bartlett tests. Differences between groups were analyzed by one-way ANOVA test, followed by Bonferroni correction, or Kruskal-Wallis test, followed by a Dunn multiple comparison post-test, as appropriate. Differences between groups for quantitative variables measured over time were analyzed by two-way ANOVA test with group and time as factors. Bonferroni correction was applied to post-hoc t-tests when ANOVA was significant. Survival curves were analyzed using Log-rank (Mantel-Cox) test. All p values were two-tailed with statistical significance indicated by a value of p < 0.05.

## Results

### Body weight and survival

Body weight increased with time in sham-operated mice (Fig. 1A,B) but this increase was higher in C57BL/6JRj mice compared to 129/Sv (ΔW0–W12: 7.9 ± 0.9 *vs* 6.1 ± 1.1 g, p < 0.01). After 5/6 Nx, body weight gradually increased in C57BL/6JRj mice but not in 129/Sv mice (ΔW0–W12: 4.7 ± 0.9 *vs* −1.9 ± 0.8 g, p < 0.001) (Supplementary Fig. 1). After 12 weeks, survival was not significantly affected by 5/6 Nx neither in C57BL/6JRj nor in 129/Sv mice.

### Kidney parameters

**Figure 2:**
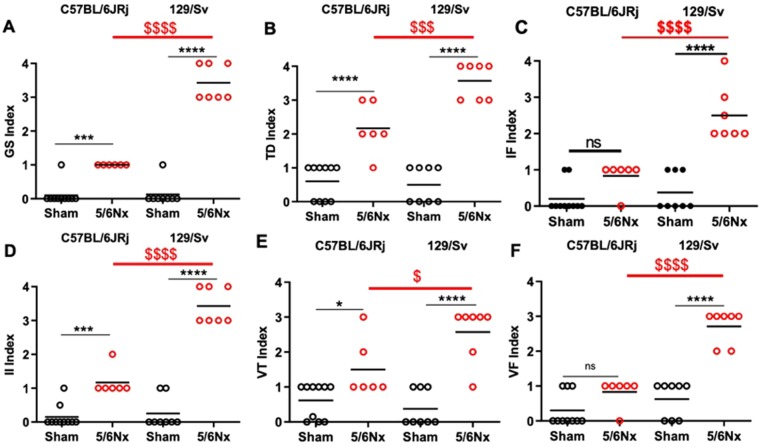
Scoring of kidney lesions 12 weeks after surgery in sham-operated and 5/6 Nx C57BL/6JRj and 129/Sv mice (n = 6–10 per group). (**A**) Glomerular sclerosis (GS) lesions, (**B**) Tubular damage (TD) lesions, (**C**) Interstitial fibrosis (IF) lesions, (**D**) Interstitial inflammation (II) lesions, (**E**) Vascular thickening (VT) lesions, (**F**) Vascular fibrosis (VF) lesions. ***p < 0.001: sham *vs* 5/6 Nx, ^$^p < 0.05: 5/6 Nx (129/Sv) *vs*. 5/6 Nx (C57BL/6JRj), ^$$$^p < 0.001: 5/6 Nx (129/Sv) *vs*. 5/6 Nx (C57BL/6JRj).

The onset of CKD was confirmed by the significant increase in plasma creatinine 12 weeks after 5/6 Nx in both strains. There was no significant interstrain difference regarding the increase in C57BL/6JRj vs 129/Sv mice (ΔW0–W12: +14 vs +28 µmol/L) (Fig. 1E). To further describe the kidney consequences of 5/6 Nx, we analyzed urine collections. As opposed to C57BL/6JRJRj mice, 129/Sv mice presented significant 2.3 and 2.8-fold increases in urine volume at week 4 and week 8 respectively (Fig. 1C,D). An increase in urinary aldosterone was observed in 129/Sv and C57BL/6JRj mice after 5/6 Nx, without difference between strains (Fig. 1F). In addition, urinary albumin/creatinine ratio was markedly increased in 129/Sv mice (4588 ± 821 *vs* 219 ± 52 mg/g, p < 0.001) but not in C57BL/6JRj mice (121 ± 29 *vs* 153 ± 14 mg/g, p = NS). Kidney histology analyses showed the development of glomerulosclerosis, tubular injury, interstitial fibrosis and inflammation, perivascular fibrosis and vascular thickening in 129/Sv and C57BL/6JRj mice after 5/6 Nx (Fig. 2A–F). All these lesions were significantly higher in 129/Sv compared to C57BL/6JRj mice.

### Blood pressure

**Figure 3:**
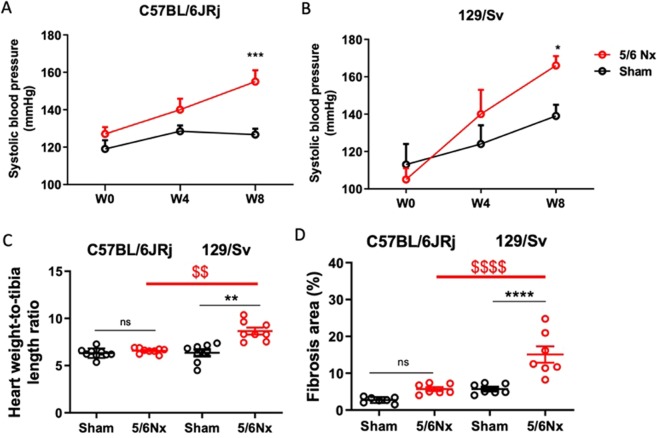
Evolution of systolic blood pressure from week 0 (W0; 5/6 Nx) to W12 (sacrifice) in sham-operated and 5/6 Nx C57BL/6JRj (**A**) and 129/SV (**B**) mice (n = 10 per group). *p < 0.05: sham *vs*. 5/6 Nx, ***p < 0.001: *vs*. 5/6 Nx. Heart weight (w) to tibia length ratio (**C**) and morphometric quantification of left ventricular cardiac fibrosis determined 12 weeks after surgery (**D**) in sham-operated and 5/6 Nx C57BL/6JRj and 129/SV mice (**B**) (n = 7–9 per group). **p < 0.01: sham *vs*. 5/6 Nx, ^$$^p < 0.01: 5/6 Nx (129/Sv) *vs*. 5/6 Nx (C57BL/6JRj), ***p < 0.001: sham *vs*. 5/6 Nx, ^$$$^p < 0.001: 5/6 Nx (129/Sv) *vs*. 5/6 Nx (C57BL/6JRj).

Systolic blood pressure was significantly increased in 129/Sv and C57BL/6JRj mice 8 weeks after surgery (Fig. 3A,B).

### Cardiac parameters

Echocardiographic measurements are presented in Table 1. There was no significant difference between sham-operated and 5/6 Nx mice in both C57BL/6JRj and 129/Sv strains regarding heart rate, LV end-diastolic diameter and wall thicknesses. However, 5/6 Nx induced a similar decrease in fractional shortening and LVEF in both strains, showing a decrease in systolic function. In addition, the E/A ratio similarly decreased after 5/6 Nx in C57BL/6JRj and 129/Sv, indicating the development of diastolic dysfunction. At sacrifice, no difference in cardiac weight was observed between 5/6 Nx and sham-operated mice in the C57BL/6JRj strain, while a slight but significant increase was noticed in 129/Sv 5/6 Nx mice (Fig. 3A). In addition, there was an increase in heart fibrosis after 5/6 Nx in 129/Sv mice, but not in C57BL/6JRj (Fig. 3B).

**Table 1:** Echocardiographic measurements in sham-operated and 5/6 Nx C57BL/6JRj and 129/Sv mice.

Mice	C57BL/6JRj		Sham *vs* 5/6 Nx	129/Sv		Sham *vs* 5/6 Nx
Parameters	Sham	5/6 Nx	p	Sham	5/6 Nx	p
HR (bpm)	404 ± 15	437 ± 12	NS	434 ± 9	429 ± 8	NS
LVEDD (mm)	4.0 ± 0.1	3.8 ± 0.1	NS	3.6 ± 0.1	3.8 ± 0.2	NS
FS (%)	39 ± 4	27 ± 3	<0.05	44 ± 3	31 ± 3	<0.05
LVEF (%)	75 ± 4	59 ± 5	<0.05	80 ± 3	64 ± 4	<0.05
E/A	1.29 ± 0.04	0.95 ± 0.03	<0.0001	1.35 ± 0.04	0.95 ± 0.03	<0.0001

Values are expressed as mean ±SEM. (n = 8–9 per group). E/A: early to late mitral inflow peak ratio, LVEF: left ventricular ejection fraction, FS: fractional shortening, LVEDD: Left ventricular end-diastolic diameter, HR: Heart rate, NS: non significant.

### Vascular function

**Figure 4:**
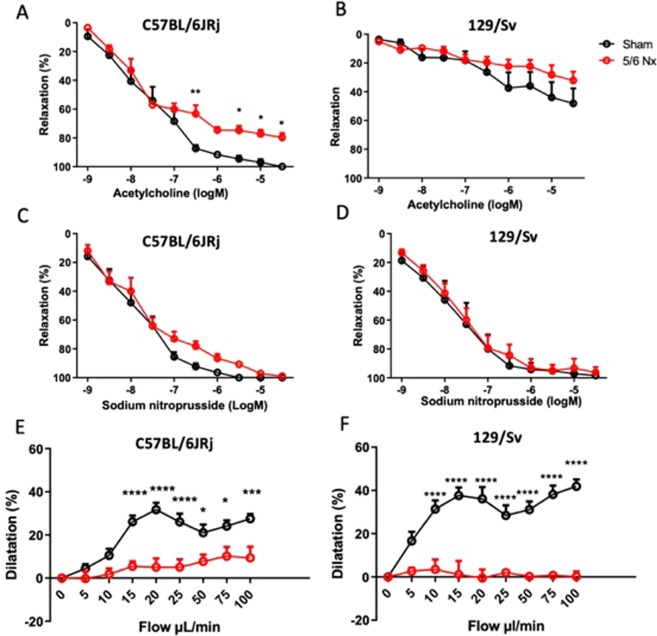
Relaxing responses of pre-contracted mesenteric arteries in response to acetylcholine (**A**,**B**) and sodium nitroprusside (**C**,**D**) in sham-operated and 5/6 Nx C57BL/6JRj (**A**,**C**) and 129/Sv (**B**,**D**) mice (n = 7–8 per group). Dilatory responses of pre-constricted mesenteric arteries in response to stepwise increase in intraluminal flow in sham-operated and 5/6 Nx C57BL/6JRj (**E**) and 129/Sv (**F**) mice (n = 7–8 per group). *p < 0.05: sham *vs*. 5/6 Nx, **p < 0.01: sham *vs*. 5/6 Nx, ***p < 0.001: sham *vs*. 5/6 Nx.

Relative to baseline values, there was a decrease of mesenteric artery relaxation to acetylcholine in C57BL/6JRj mice compared to sham-operated mice at week 12, without change in the relaxation response to sodium nitroprusside (SNP), demonstrating endothelial dysfunction (Fig. 4A,C). No difference was shown regarding mesenteric artery relaxation to acetylcholine and SNP in 129/Sv mice compared to sham-operated mice (Fig. 4B,D). Importantly, the relaxation to acetylcholine was altered in sham-operated 129/Sv mice (Fig. 4B), demonstrating that 129/Sv mice do not present the “normal” response to acetylcholine (acetylcholine concentration-dependent relaxation of the artery related to the release of endothelium-derived vasodilators).

Regarding arteriography, basal external diameter of the mesenteric artery increased after 5/6 Nx in C57BL/6JRj (Sham-5/6 Nx: 301 ± 10 *vs* 348 ± 4 µm, p = 0.001) but not in 129/Sv mice (Sham-5/6 Nx: 277 ± 14 *vs* 291 ± 13 µm, p = NS). Wall thickness increased significantly after 5/6 Nx in both C57BL/6JRj (Sham-5/6 Nx: 21 ± 1 *vs* 32 ± 2 µm, p < 0.001) and 129/Sv (Sham-5/6 Nx: 22 ± 1 *vs* 31 ± 1 µm, p < 0.001) strains. In addition, in precontracted mesenteric arteries we observed a marked decrease of the endothelium-dependent vasodilation in response to a gradual increase in intraluminal flow in both C57BL/6 JRj (Fig. 4E) and 129/Sv (Fig) mice, demonstrating severe endothelial dysfunction in both strains.

## Discussion

The precise cardiovascular consequences of the 5/6 Nx model are poorly defined in mice, because of methodological differences between studies (Table 2). The present study demonstrates that 5/6 Nx is a relevant tool to study the cardiovascular consequences of CKD in both C57BL/6JRj and 129/Sv mice. Importantly, it highlights striking differences in terms of kidney, cardiac and vascular consequences of this model between strains, with important implications for the design and interpretation of experimental procedures.

**Table 2:** Studies investigating kidney, cardiac and vascular consequences of 5/6 Nx in C57BL/6JRj and 129/Sv mice.

	Studies	Methods	Polyuria	Urine albumin	Plasma aldosterone	HTN	Cardiac hypertrophy	Cardiac fibrosis	Systolic function	Diastolic function	Endothelial function
C57BL/6JRj	Leelahavanichkul A *et al*. 2010	surgical removal	ND	−	ND	−	ND	−	ND	ND	ND
	Ma L-J *et al*. 2003	surgical removal	ND	−	ND	−	ND	ND	ND	ND	ND
	Li Y *et al*. 2009	surgical removal	ND	ND	ND	+	+	+	+	+	ND
	Kobayashi R *et al*. 2017	surgical removal	+	ND	−	−	ND	ND	ND	ND	ND
	Wei J *et al*. 2018	surgical removal	ND	−	−	−	ND	ND	ND	ND	ND
	Wei J *et al*. 2018	artery ligation	ND	+	−	+	ND	ND	ND	ND	ND
	Gava AL *et al*. 2012	surgical removal	+	+(proteinuria)	ND	+	ND	ND	ND	ND	ND
	Lehners A *et al*. 2014	surgical removal	ND	+	ND	−	−	ND	ND	ND	ND
	Jin J *et al*. 2017	surgical removal	ND	ND	ND	−	ND	ND	ND	ND	+kidney artery
	Thomsen MB *et al*. 2018	surgical removal	ND	ND	ND	ND	+	ND	ND	ND	ND
	Madsen M *et al*. 2017	surgical removal	ND	ND	ND	ND	ND	ND	ND	ND	−aortic ring
129/Sv	Leelahavanichkul A *et al*. 2010	surgical removal	ND	+	ND	+	ND	+	ND	ND	ND
	Ma L-J *et al*. 2003	surgical removal	ND	+	ND	+	ND	ND	ND	ND	ND
	Winterberg PD *et al*. 2016	surgical removal	ND	ND	ND	+	+	+	−	+	ND
	Kobayashi R *et al*. 2017	surgical removal	+	ND	ND	+	ND	ND	ND	ND	ND
	Jung O *et al*. 2010	artery ligation	ND	+	ND	+	ND	ND	ND	ND	ND

(−) no significant difference or (+) significant difference between 5/6 Nx and control mice; (ND) not determined.

In line with results of the present study, Leelahavanichkul *et al*. previously reported that C57BL/6JRj mice presented milder CKD than 129/Sv and CD-1 mice after subtotal nephrectomy^17^. Accordingly, we found that in the remnant kidney of C57BL/6JRj mice tubular damage, interstitial fibrosis and inflammation were less severe compared to 129/Sv mice. C57BL/6JRj did not develop albuminuria while 129/Sv did, which is consistent with previous findings by Ma LJ *et al*.^21^. This was associated with increased glomerulosclerosis in 129/Sv mice in both studies.

Concerning cardiac consequences of CKD, in our study both strains presented diastolic dysfunction with a mild decrease in systolic function. This can be related to cardiac hypertrophy and remodelling, leading to heart wall stiffness, impairing relaxation. As opposed to C57BL/6JRj, 129/Sv exhibited hypertrophy, and presented increased cardiac fibrosis. Siedlecki *et al*.^22^ observed cardiac hypertrophy in 129/Sv mice with modified nephrectomy (induced by cauterization of one kidney and total nephrectomy of the other one). To our knowledge, no cardiac hypertrophy after 5/6 Nx has been reported in C57BL/6JRj to date. In C57BL/6JRj, 8 weeks after 5/6 Nx Han *et al*.^23^ showed diastolic dysfunction and cardiac fibrosis, and 12 weeks after 5/6 Nx Li *et al*.^24^ described systolic and diastolic dysfunction, with increased cardiac fibrosis. In 129/Sv, 16 weeks after 5/6 Nx Winterberg *et al*.^25^ showed cardiac fibrosis, diastolic dysfunction without systolic dysfunction.

The pathophysiology of the cardiac consequences of CKD involves multiple factors, including hypertension, uremic toxins, FGF-23, and activation of the renin angiotensin aldosterone system^26–28^. We found an increase of urinary aldosterone excretion after 5/6 Nx, which was similar between strains. Kobayashi *et al*.^29^ compared 5/6 Nx in C57BL/6JRj and 129/Sv mice and found no difference in plasma aldosterone between strains after 5/6 Nx. Differential expression of angiotensin II type 1 receptor-associated protein may explain the differential activity of the renin angiotensin aldosterone system, independently of plasma and urine aldosterone concentration. As is frequently the case in human, cardiac hypertrophy was associated with albuminuria in 129/Sv mice, while no albuminuria was observed in C57BL/6JRj^2,30,31^.

Hypertension is frequently encountered in kidney diseases. As is also the case in experimental models of diabetes, strain differences have an impact in murine models of hypertension^32^. Although the gold standard method to measure blood pressure in mice is telemetry, controversy exists and the tail-cuff method, which we report here, provides valuable information within normal to high-normal values of blood pressure^33,34^. Previous studies have reported hypertension in 129/Sv after 5/6 Nx, both by plethysmography with tail cuff^35^ and by telemetry^17^, contrasting with the C57BL/6JRj strain in which blood pressure most frequently does not increase after 5/6 Nx^21^. The increase in SBP we describe in C57BL/6JRj with our model of 5/6 Nx therefore contrasts with existing literature. Wei *et al*. compared two models of 5/6 Nx in C57BL/6JRj mice, one with subtotal surgical removal and the other, as in our study, using the ligature of the upper branch of kidney artery, without removing the ischemic kidney parenchyma^36^. Hypertension was described only in the latter model. Wei *et al*. found that this model upregulates kidney and systemic inflammatory response, which likely promotes the development of hypertension.

Impaired endothelial function has been extensively studied in patients with CKD, and is considered as a major player in the detrimental interactions between the kidney and the cardiovascular system in CKD^37^. Endothelial dysfunction after 5/6 Nx has previously been shown in rat models of CKD^38^. In mice models of CKD impaired flow-mediated vasodilation in a resistance vessel had not been reported previously, while this is generally considered as a gold-standard method to demonstrate and study endothelial dysfunction. We demonstrated endothelial dysfunction after 5/6 Nx in both strains. Previous studies have shown features of impaired endothelial function in CKD models by means of dysregulated biomarkers. D’Apolito *et al*.^39^ have shown impaired dilation of the thoracic aorta to acetylcholine in C57BL/6JRj mice after 5/6 Nx, with a myograph-based analysis.

129/Sv mice are more prone to CV consequences of CKD than C57BL/6JRj mice. It is therefore important to highlight that in 129/Sv mice the *ex vivo* analysis of resistance arteries can only be performed by arteriograph evaluation of flow-mediated dilatation and not myograph evaluation of the response to acetylcholine, since this response was incomplete in control 129/Sv mice when compared to other strains. Although data in the literature are scarce, Liu *et al*.^40^ had already showed incomplete relaxation to acetylcholine in mesenteric artery in 129/Sv mice. Ryan *et al*.^41^ found similar results on aorta in 129/Sv mice but complete relaxation to acetylcholine when testing carotid artery. One of the explanations is a possible decrease of acetylcholine receptor in abdominal arteries, especially since the use of other endothelium-dependent agonists on the aorta induced similar relaxation as compared to C57BL/6JRj. No smooth muscle cell dysfunction was found after 5/6 Nx in our study, contrasting with increased wall thickness in both strains. The increase in wall thickness is mainly due to media remodeling, with hypertrophy and increased fibrogenesis of smooth muscle cells. Results in our study suggest that the remodeling in this model is not sufficient not to alter smooth muscle cell reactivity (precontraction and response to SNP).

The genome and the genetic differences between C57BL/6J and 129/Sv mice are available in large databases (http://bioit2.irc.ugent.be/prx/mousepost/Home.php), (http://www.informatics.jax.org/home/strain)^42^. Over 8800 significant genetic differences (insertions, deletions, mutations, SNPs) with a potential effect on protein function exist between the 2 strains compared in our study. The database published by Timmermans *et al*. identifies several candidate genes playing a role in vascular physiology (*Ren1, Angpt4, Notch1*) or inflammation and fibrogenesis (*Ddr1, Mmp9, Col6A5, Smad7*) which may contribute to the differences observed in the present study^42^. Further studies on candidate genes responsible for the variable susceptibility of these 2 strains to 5/6 Nx could suggest targets to limit the CV consequences of CKD.

In conclusion this study comparatively details kidney and cardiovascular consequences of the 5/6 Nx model of CKD in two of the most frequently studied strains: C57BL/6JRj and 129/Sv male mice. 5/6 Nx induced higher kidney injury, albuminuria, hypertension, cardiac hypertrophy and fibrosis, diastolic and systolic dysfunction and endothelial dysfunction in 129/Sv. In C57BL/6JRj while mild kidney injury was observed, no albuminuria, cardiac hypertrophy or fibrosis were present. These results are of importance when comparing conclusions of the literature in different strains. They will also be particularly useful for scientists to provide arguments defining whether C57BL/6JRj or 129/Sv mice should be used to investigate specific research issues on the CV and kidney consequences of CKD.

## Supplementary information

Supplementary results.
