# Supplementary material for: 5/6 nephrectomy induces different renal, cardiac and vascular consequences in 129/Sv and C57BL/6JRj mice
Source: Sci Rep. 2020 Jan 30;10:1524. doi: 10.1038/s41598-020-58393-w (PMC6992698; doi:10.1038/s41598-020-58393-w)
Supplement: Supplementary file 1 — Supplementary results. [file 41598_2020_58393_MOESM1_ESM.docx]

**5/6 nephrectomy induces different renal, cardiac and vascular**

**consequences in 129/Sv and C57BL/6JRj mice**

AUTHORS:

Mouad Hamzaoui ^1,2^, *MD, MSc*, Zoubir Djerada ^1,3^, *PharmaD, PhD*, Valery Brunel ^4^, *PharmD,* Paul Mulder ^1^, *PharmD, PhD*, Vincent Richard ^1^, PhD, Jérémy Bellien ^1,5^, *PharmD, PhD*, Dominique Guerrot ^1,2^, *MD, PhD.*

AFFILIATIONS:

1. Normandie Univ, UNIROUEN, INSERM U1096, FHU REMOD-VHF, 76000 Rouen, France
2. Nephrology Department, Rouen University Hospital, Rouen, France
3. Pharmacology Department, Reims University Hospital, Reims, France
4. Biochemistry Department, Rouen University Hospital, Rouen, France
5. Pharmacology Department, Rouen University Hospital, Rouen, France

CORRESPONDING AUTHOR:

Prof. Dominique Guerrot

Nephrology Department, Rouen University Hospital, Rouen, France

1 rue de Germont, 76031 Rouen, France

[dominique.guerrot@chu-rouen.fr](mailto:dominique.guerrot@chu-rouen.fr)

ORCID : 0000-0002-5953-5785

Tel: +33 2 32 88 90 02

Fax: +33 2 32 88 91 15

**Supplementary information**


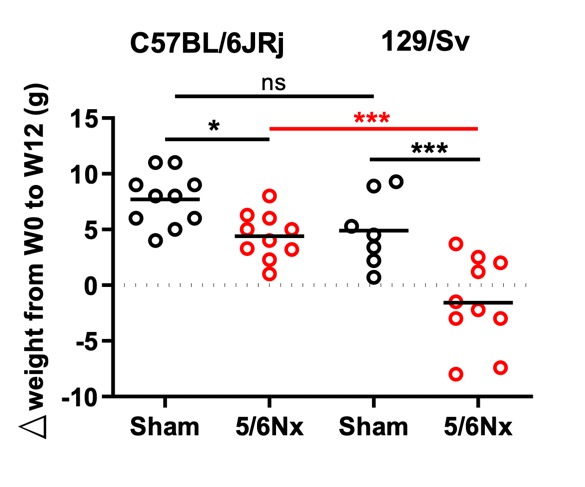


**Supplementary Figure 1.** Weight evolution between week 0 and week 12 in C57BL/6JRj and 129/Sv mice (n=7-10 per group). *p<0.05: sham vs. 5/6 Nx, ***p<0.001: sham vs. 5/6 Nx, ***p<0.001: 5/6 Nx C57BL/6JRj vs. 5/6 Nx 129/Sv.


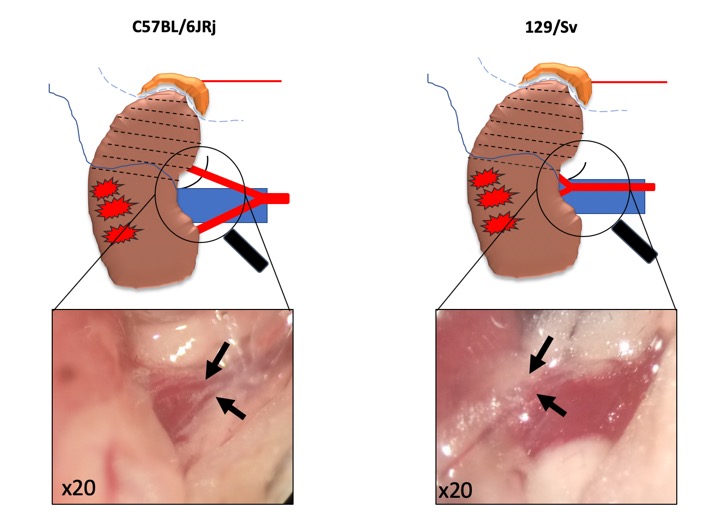


**Supplementary Figure 2.** Schematic view and representative photography of the characteristic division of the kidney artery at the hilum in C57BL/6JRj(left) and 129/Sv (right) mice.


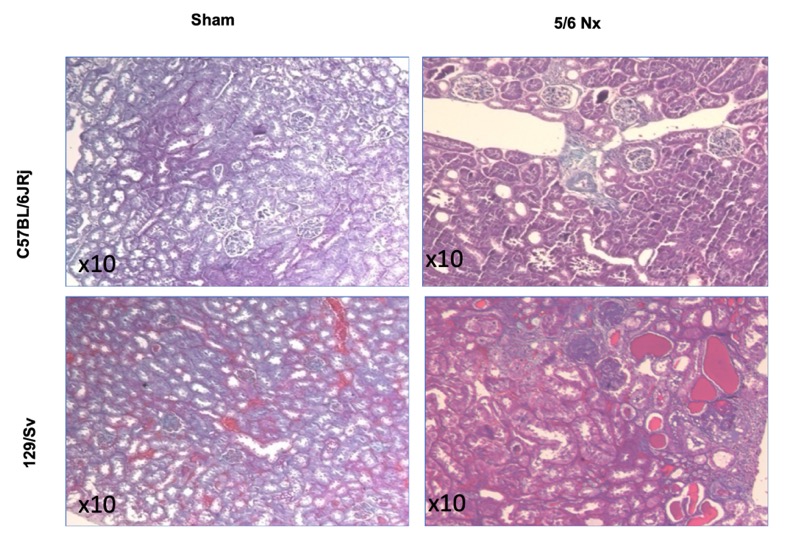


**Supplementary Figure 3.** Representative views of kidney histology after Masson staining at magnification X10 12 weeks after surgery in sham-operated and 5/6 Nx C57BL/6JRj and 129/Sv mice.


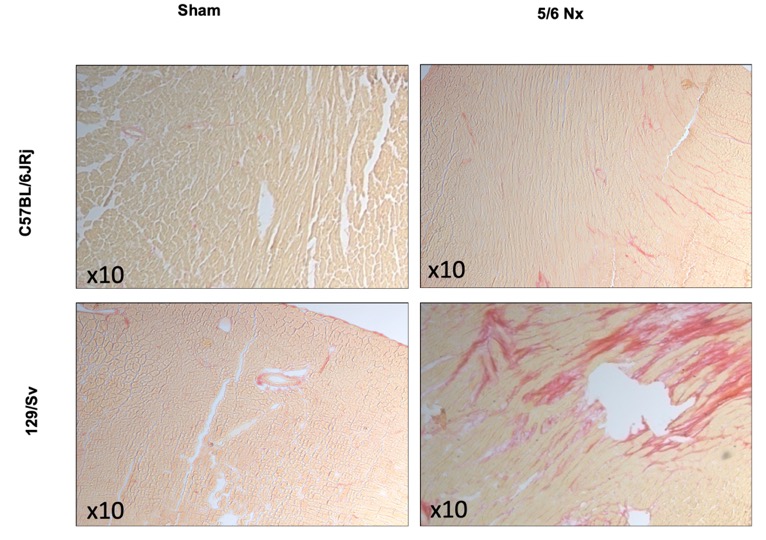


**Supplementary Figure 4.** Representative views of heart section after red Sirius staining at magnificence x10 12 weeks after surgery in sham-operated and 5/6 Nx C57BL/6JRj and 129/Sv mice.
